# A Much Needed Work

**Published:** 1891-03

**Authors:** N. B. Warren

**Affiliations:** Hazel Dell, Miss.


### Correspondence.

### A MUCH NEEDED WORK.

*Editors So. Med. Record:*

While every branch of medicine has made, and is still making such rapid strides towards perfection, there is one branch, or at least a work closely connected with medicine, that has been, so far as I know, generally neglected. And in my opinion the profession will continue to be crippled in its work, until it is taken in charge by men of science, and placed on its proper footing. I refer to collecting vegetable herbs.

A great many of our valuable drugs are obtained from herbs found in foreign countries—some are found in the tropics—and in nearly all cases people who know nothing more than the general external apperance of the herbs, are entrusted with gathering them. There is often two or more varieties so nearly alike in external appearance that it is difficult to distinguish them, while their medicinal properties are very different. Barks can nearly always be counterfeited. Again, there is a certain time herbs should be gathered. These collectors go to work when it is most convenient; their motto being "quantity, not quality."

The herbs gathered are packed in a most careless manner, and put on the market; where they sometimes remain for years. During this time they may be subjected to all kinds of exposures, and lose a large part of their medicinal properties. What we need is botanical gardens established in every climate, and competent men put in charge; and the cultivation of all valuable medical herbs conducted on scientific principles. This done, and I think a few years would see a marked change for the better in the doctors' medical success. This will soon become a necessity in some cases, as some herbs are becoming scarce, and if not cultivated will soon not supply the demand.

I believe that some of our best medicines are found in the vegetable kingdom, but until more care is taken in obtaining them, we may better rely on mineral drugs, which are less abused in their preparation.

N. B. WARREN, M. D.

Hazel Dell, Miss.

